# Uterine rupture in the first trimester: a case report and review of the literature

**DOI:** 10.1186/s13256-023-04318-w

**Published:** 2024-01-06

**Authors:** Steve Kyende Mutiso, Felix Mwembi Oindi, Debbie Muthoni Mundia

**Affiliations:** Department of Obstetrics and Gynaecology, Aga-Khan University, P.O. Box 30270-00100, Nairobi, Kenya

**Keywords:** Uterine dehiscence, First trimester, Outcome, Complications

## Abstract

**Background:**

Uterine rupture is a rare complication that can occur in the first trimester of pregnancy. It can lead to serious maternal morbidity or mortality, which is mostly due to catastrophic bleeding. First trimester uterine rupture is rare; hence, diagnosis can be challenging as it may be confused with other causes of early pregnancy bleeding such as an ectopic pregnancy. We present a case of first trimester scar dehiscence and conduct a literature review of this rare condition.

**Case presentation:**

A 39-year-old African patient with four previous hysterotomy scars presented with severe lower abdominal pain at 11 weeks of gestation. She had two previous histories of third trimester uterine rupture in previous pregnancies with subsequent hysterotomies and repair. She underwent a diagnostic laparoscopy that confirmed the diagnosis of a 10 cm anterior wall uterine rupture. A laparotomy and repair of the rupture was subsequently done.

**Conclusion:**

In conclusion, the case presented adds to the body of evidence of uterine scar dehiscence in the first trimester. The risk factors, clinical presentation, diagnostic imaging, and management outlined may help in early identification and management of this rare but life threatening condition.

## Background

Uterine rupture refers to the complete disruption of uterine layers leading to changes in fetal and maternal status [1]. It carries a high risk of both maternal and fetal morbidity and mortality [1]. Uterine rupture in the first trimester of pregnancy is very rare and the actual incidence in literature is unclear [2]. Most cases of uterine rupture occur in the second and third trimester [3]. Globally, the incidence of uterine rupture is 0.07%, while in Africa the incidence is 1.3% [4].

The most common risk factor for uterine rupture is a previous cesarean section scar especially when attempting a vaginal birth after a cesarean section [1]. The risk is increased with short interpregnancy interval, a classical uterine scar, and administration of misoprostol. [5] The incidence of rupture in women with a previous cesarean section scar is 0.3% [6] while that of an unscarred uterus is 1 in 5700 to 1 in 20,000 pregnancies [7]. Other risk factors include a previous myomectomy scar and dilation and curettage. Major abdominal trauma occurring during pregnancy such as a motor vehicle accident or a fall can also lead to uterine rupture [8].

The classical symptoms of uterine rupture are acute severe abdominal pain and vaginal bleeding. The patient may present with hemodynamic instability with hypotension and tachycardia. Hypotension may present as dizziness, nausea, vomiting, and lightheadedness. Infrequently, bladder injury may also occur presenting with hematuria [9]. On examination, the abdomen is peritonitic and irritation of the diaphragm by the blood in the peritoneum would lead to referred shoulder tip pain. Intrapartum monitoring may reveal sudden fetal bradycardia, loss of fetal station, and variable decelerations and decreased contraction pattern [10]. Complications of uterine rupture include hemorrhage, shock, bladder injury, and maternal and fetal death.

Timely diagnosis and intervention is essential in managing these patients. A focused abdominal sonography for trauma can be done to ascertain the diagnosis and rule out differential diagnosis such as an ectopic pregnancy. Typical findings are free fluid in the peritoneum, an abnormality in the uterine wall, or fetal parts outside the uterus [11].

Management of uterine rupture involves surgery to control maternal hemorrhage. The treatment depends on the location of uterine rupture, the degree of involvement of parauterine tissue, and desire for subsequent pregnancy. This includes uterine repair or hysterectomy in the case of severe uterine rupture [9]. The recommended approach to repair is exploratory laparotomy rather than minimally invasive surgery [12]. Most women will survive uterine rupture with prompt surgical intervention and resuscitation. The risk of recurrent uterine rupture after previous repair is not well known as the general incidence of uterine rupture is low. [13].

We describe a rare case of first trimester uterine dehiscence and conduct a subsequent literature review of this disease.

## Case presentation

### Patient information

A 39-year-old African para 4 + 1, gravida 6 presented at 11 weeks of gestation with severe lower abdominal pain that was sharp and continuous in character and had gradually worsened over the last 4 h. She had associated dizziness but no vaginal bleeding. She had a history of four previous hysterotomy scars with only one living child. She had two previous uterine ruptures in 2016 and 2019 at 34 weeks and 28 weeks, respectively. Her obstetric history revealed the following: her first pregnancy was in 2013, which was complicated by an emergency cesarean section (CS) at 28 weeks due to non-reassuring fetal status and subsequently a neonatal death shortly after. The second pregnancy was in 2015, which was complicated with preeclampsia with severe features; the patient had an emergency CS at 31 weeks and a neonatal death thereafter. Her third pregnancy was in 2016, she developed preeclampsia and was delivered at 36 weeks via emergency CS due to uterine rupture with a good neonatal outcome. Her fourth pregnancy in 2018 ended up in an incomplete miscarriage in the first trimester that was medically managed. Her fifth pregnancy in 2019 ended up with a uterine rupture and a stillbirth at 28 weeks of gestation.

Antenatally, the patient had been positively screened for antiphospholipid syndrome and was started on subcutaneous enoxaparin 40 U daily and acetyl-salicylic acid (asprin) 150 mg (mg) daily. Her antenatal profile, dating scan, and nuchal translucency were all normal.

### Clinical findings

On admission, her vitals were stable with blood pressure of 110/67 mmHg, pulse rate of 89 beats per minute, oxygen saturation of 100%, respiratory rate of 16 breaths per minute, and temperature of 36.8 °C. She had mild conjunctival pallor and on abdominal examination fundal height corresponded to a 15-week gestation with generalized severe tenderness with guarding.

### Diagnostic assessment

Her complete blood count showed hemoglobin of 11.2 g/dl, platelets of 226 cells/µl and white blood cells of 15.29 cells/µl.

**Fig. 1:**
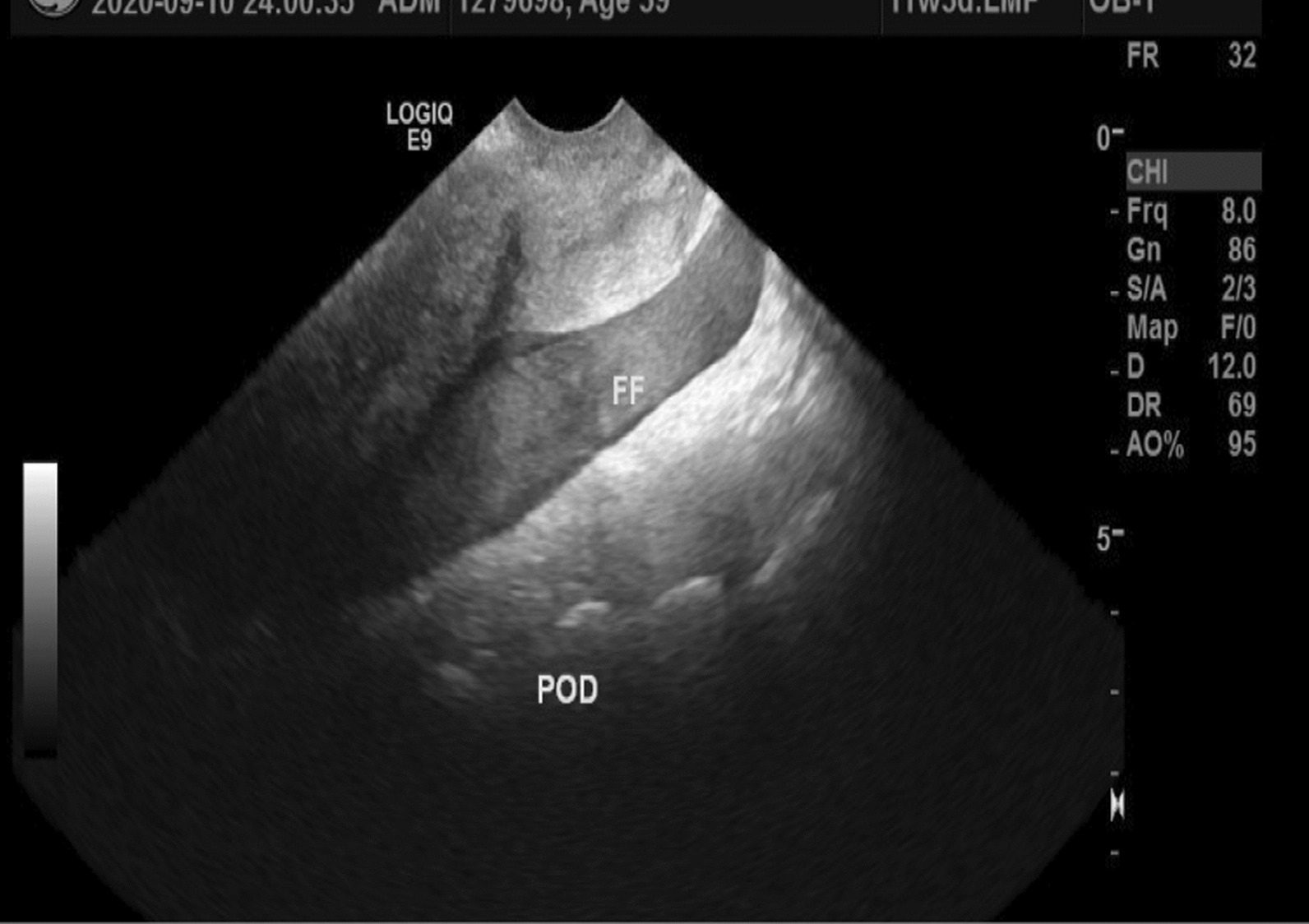
Free fluid in the pouch of Douglas. FF, free fluid; POD, pouch of Douglas

**Fig. 2:**
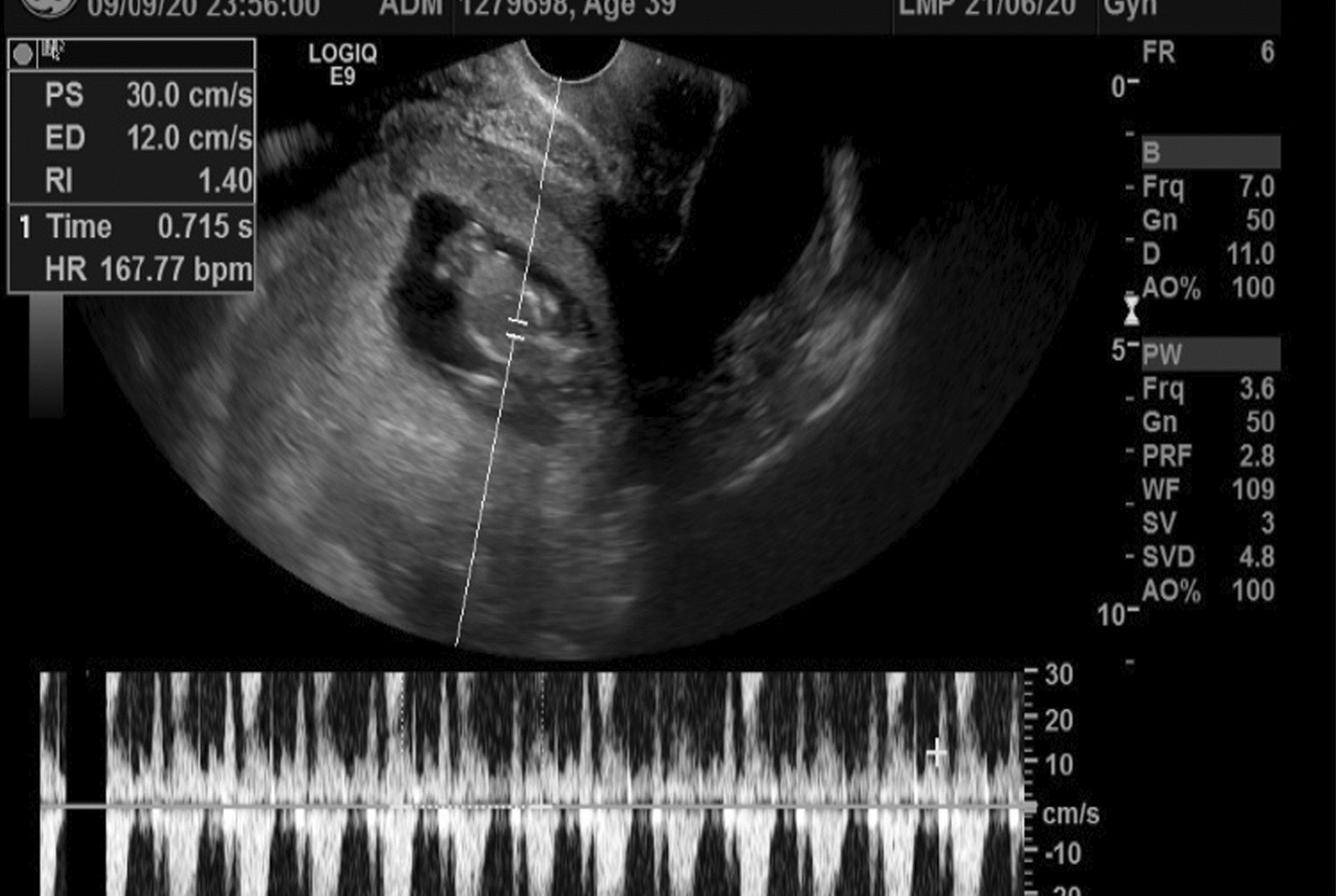
Intrauterine pregnancy with cardiac activity

A focused abdominal sonogram of trauma (FAST) ultrasound revealed free fluid in the abdomen with a viable intrauterine pregnancy (Figs. 1, 2). She consented to an urgent diagnostic laparoscopy and was wheeled to theater immediately.

### Therapeutic intervention

**Fig. 3:**
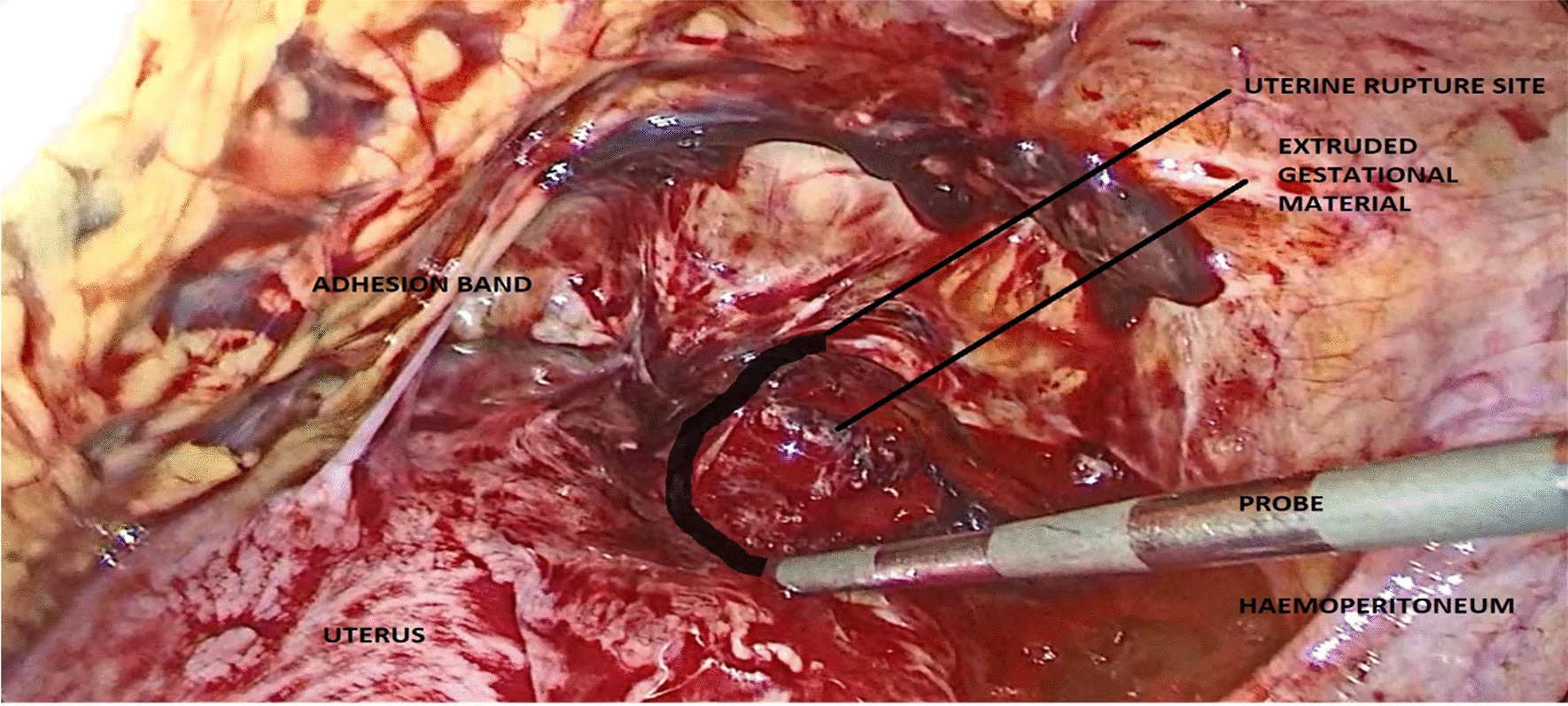
Laparascopic view showing the uterine rupture site and hamoperitoneum

**Fig. 4:**
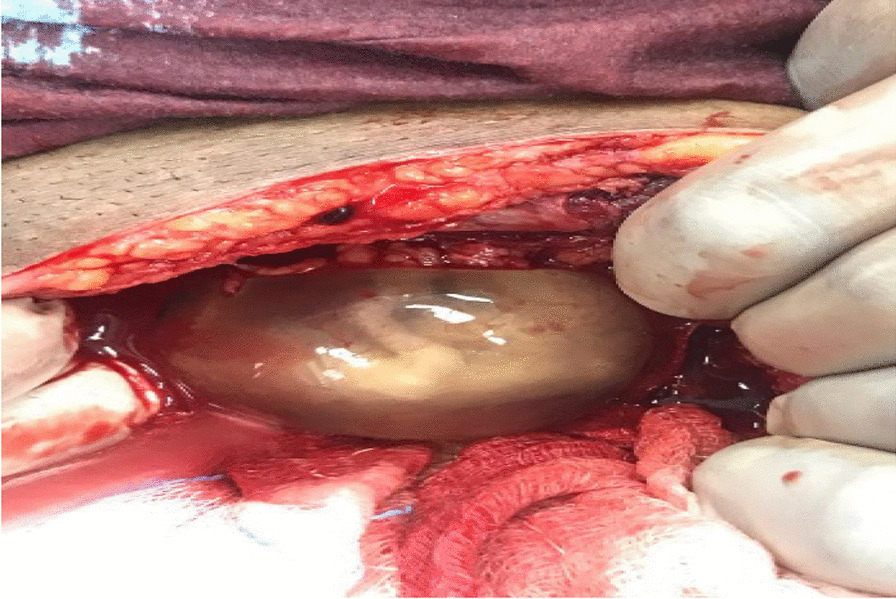
The gestational sac protruding

**Fig. 5:**
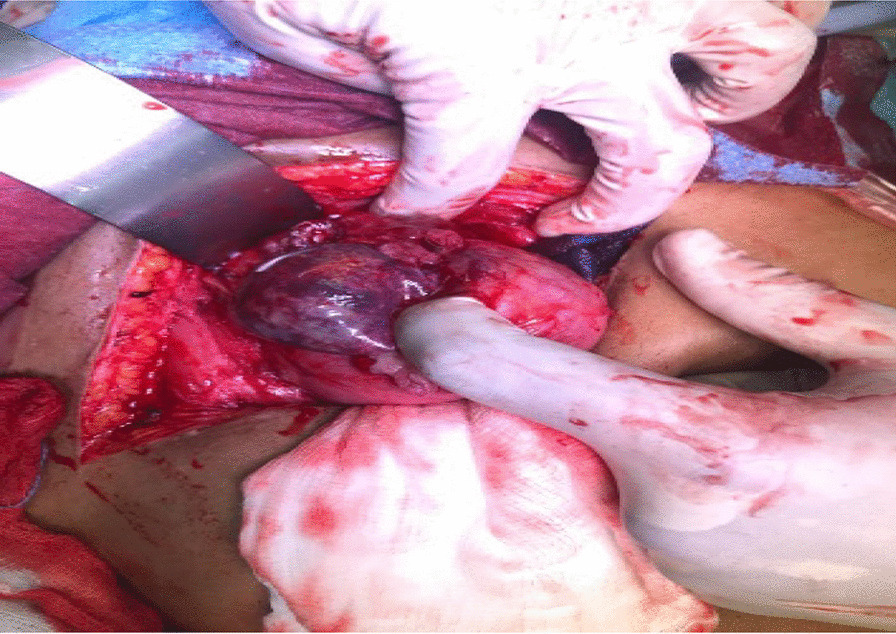
The placenta being delivered

**Fig. 6:**
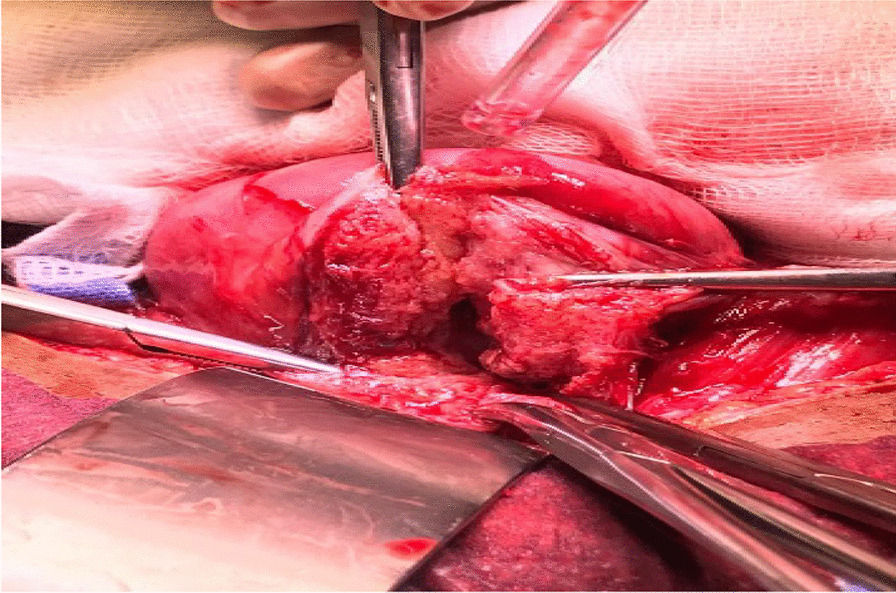
Uterine rupture approximately 10 cm in length

**Fig. 7:**
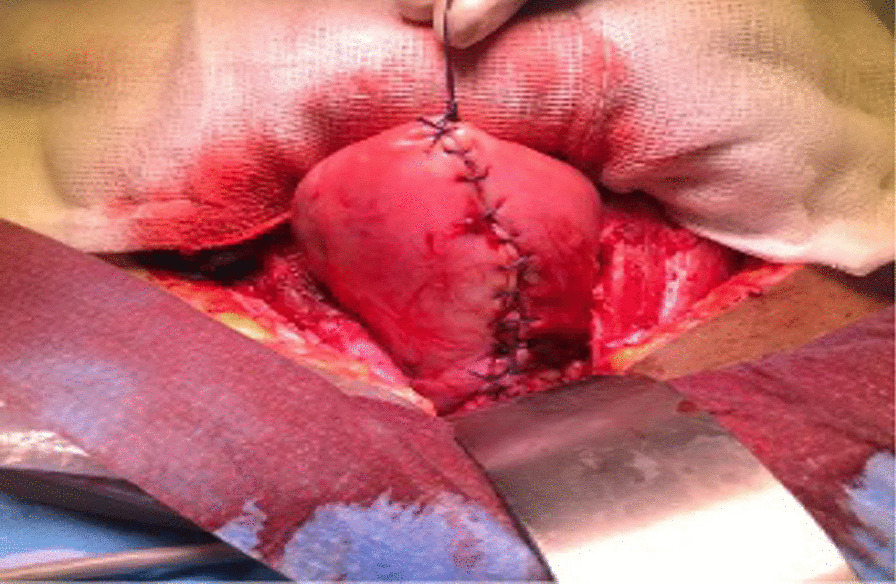
The uterus after repair

Intraoperatively, an anterior uterine rupture of approximately 10 cm was found (Fig. 3) with a hemoperitoneum of approximately 1000 ml. The tubes and ovaries appeared normal bilaterally. The decision to convert to an open procedure was made on the basis of achieving a better uterine repair due to the early nature of her dehiscence. The procedure was converted to open surgery where the gestational sac was found protruding from the uterus and was expelled spontaneously (Fig. 4). The placenta was expelled next and was noted to be attached on the posterior uterine wall (Fig. 5). The uterine rupture (Fig. 6) was repaired in three layers with polyglactin (vicryl) number 0 suture and hemostasis was achieved (Fig. 7). An abdominal washout was done with normal saline and abdomen was closed in layers. There were no intraoperative complications. She recovered well and was discharged on the third postoperative day.

## Discussion and conclusion

The above case documents the rare occurrence of uterine rupture in the first trimester. Uterine rupture is a rare condition with an incidence of approximately 0.3% during term deliveries and virtually unheard of in the first trimester [6]. It has significant associated morbidity and mortality, with mortality occurring in about 1 in 500 cases of rupture [4]. About 23% of patients with uterine ruptures will require hysterectomy with a larger proportion requiring transfusion [4]. Early detection and intervention is of great importance to avert this morbidity and mortality [9].

Uterine rupture is mostly associated with previous uterine scarring from procedures such as a prior cesarean section, myomectomy, or hysteroscopic resection of a uterine septum [14]. Although data is limited, a history of previous rupture significantly impacts on the incidence of uterine scar dehiscence in subsequent pregnancies [1]. Most cases of uterine rupture occur during labor with a myriad of predisposing factors identified [3]. These include induction of labor with misoprostol, which has been shown to have a uterine rupture risk of 5–10% when used in patients with a previous cesarean delivery [6]. Obstructed labor or labor dystocia at advanced dilation (greater than 7 cm) and a prolonged second stage have also been associated with an increased rupture risk [6]. Other associated factors include fetal manipulation during delivery with procedures such as forceps delivery and internal podalic version being shown to increase the risk of rupture [1]. Malpresentation and abnormal placentation such as the placenta accrete spectrum have also been shown to impact on uterine rupture [3]. Iatrogenic causes of uterine rupture include instrumentation during evacuation of a miscarriage [15].

Only a few cases are documented in literature on spontaneous uterine rupture in the first trimester. These cases have had a myriad of predisposing factors reported with one being a case of placenta percreta-induced uterine rupture in the first trimester in a patient who had no previous history of cesarean section but had two previous spontaneous abortions treated by dilation and curettage [16]. Another case documents a 36-year-old para 2 + 0 who had a spontaneous uterine rupture at 10 weeks in a non-scarred uterus with no predisposing factors [3]. Uterine anomaly has also been identified as a risk factor for uterine rupture with a reported a case of first trimester spontaneous rupture in a multigravida with a bicornuate uterus [17]. The case presented had had four previous hysterotomy scars as a predisposing factor; this was probably the most significant factor in this case. She had a normal non-invasive placentation and had no uterine anomaly.

The clinical presentation of a uterine rupture in the first trimester might be nonspecific leading to delay in diagnosis [18]. This delay in diagnosis can lead to catastrophic bleeding and death. A high index of suspicion is therefore important especially in the presence of acute abdominal pain and unstable vitals. Differential diagnosis include an ectopic pregnancy, heterotopic pregnancy, molar pregnancy with molar invasion, or a bleeding corpus luteum [17]. In the present case, diagnosis was promptly made due to the patients’ previous history of uterine rupture, which gave the clinicians a high index of suspicion.

The diagnosis of uterine rupture in the first trimester can be made by ultrasound imaging [11]. The feature observed on ultrasound in this case was free fluid in the peritoneum with an intrauterine gestation. These features are the most commonly observed on sonography in cases of uterine rupture [11]. Other features that have been described include: distortion of the uterine wall or presence of fetal parts outside the uterus [11]. Features that could distinguish first trimester uterine rupture from an ectopic pregnancy include presence of an adnexal mass with a discernable gestation sac with or without cardiac activity [19]. In cases of catastrophic bleeding an ultrasound might have limited value and urgent diagnostic surgery which may be laparoscopy may be needed to diagnose and promptly treat the condition [17].

Management options for uterine rupture include performing a surgical repair of the tear or a hysterectomy when repair fails [20]. Laparotomic repair of uterine rupture has been shown to be superior to laparascopic repair due to better mobility of the surgeon in performing multilayer repair, which improves the strength of the wound, creates better exposure, and is quicker in achieving hemostasis [21, 22]. However, laparoscopic repair has been described in literature in the early postpartum period following vaginal birth after a prior cesarean [22] The advantages of this approach include quicker recovery time and a smaller surgical wound. However, its downfall is reduced exposure in performing the repair and the eventual structural integrity of the repair seems to be weaker than in laparotomic repair [22]. Diagnostic laparoscopy was performed first to ascertain the diagnosis in the present case. Laparoscopy is widely used in pregnant women for differentials of acute abdomen in the first trimester as was done in the present case [16]. This was followed by an open surgery for the repair, which was deemed as the best approach to facilitate a better structural repair since the patient was keen on future conception.

The complication of uterine rupture in the first trimester is life threatening maternal hemorrhage, which could lead to hemorrhagic shock, coagulopathy, multiorgan system failure, and eventually death [23]. Uterine rupture accounts for 14% of all hemorrhage-related maternal mortality [24]. The present case had a good outcome since quick diagnosis was made and timely repair was done to stabilize the mother.

The recurrence risk of uterine rupture in literature is estimated to be 4–33% in subsequent pregnancies[25]. Because of this, patients are advised to undergo a cesarean section in future pregnancies before the onset of labor or immediately at the onset of spontaneous preterm labor [17]. Ideal management of pregnancy after uterine rupture in first trimester has not been developed due to insufficient literature [26], but it can be posited that close clinical surveillance will aid in early identification of subsequent ruptures.

In conclusion, the case presented adds to the body of evidence of uterine scar dehiscence in the first trimester. The risk factors, clinical presentation, diagnostic imaging, and management outlined may help in early identification and management of this rare but life threatening condition.

## Data Availability

The materials used during the current study are available from the corresponding author on reasonable request.
